# Gentamicin induced Anaphylaxis, a case report

**DOI:** 10.1186/1939-4551-8-S1-A269

**Published:** 2015-04-08

**Authors:** Catalina María Gómez Henao, Cristina Isabel Herrera Morales, Ricardo Cardona Villa, Ana Maria Celis Henao

**Affiliations:** 1 Universidad De Antioquia, Colombia; 2 I.P.S Universitaria-Clinica León XIII, Colombia

## Introduction

The aminoglycosides are a group of antibiotics widely used, adverse reactions to these drugs have frequently been reported, mainly toxic (ototoxicity and nephrotoxicity); hypersensitivity reactions that occur more often are contact dermatitis, generally to ophthalmic and otic Neomycin and Gentamicin preparations, however, hypersensitivity reactions mediated by IgE, have been recently reported, founding in the literature only a few case reports of these.

## Objective

Present a case of type I hypersensitivity reaction to Gentamicin.

## Methods

A systematic literature research was conducted in databases PubMed, Embase and Lilacs, using keywords: Anaphylaxis and Gentamicin, Gentamicin and Hypersensitivity and Anaphylaxis Aminoglycosides; founding just three case reports of anaphylaxis induced by Gentamicin in the literature; then one case report of a patient with type I hypersensitivity reaction after administration of gentamicin Intramuscular, attended in allergy service in Medellin-Colombia is performed

## Results

We report a case of a female patient, 53 years old, referred to the allergy clinic service with an adverse reaction after application of gentamicin IM for clinic study. The patient was in treatment, for a superinfected third degree burn, with intramuscular gentamicin once daily for 8 days. One day after the last dose presented urticaria with angioedema and respiratory distress. After two months of these symptoms skin tests were performed with Gentamicin, prick test with a concentration of 40 mg/ml with negative reading after 20 minutes, positive control to histamine. Subsequently Intradermal test was performed with a concentration of 0,2 mg/ml with positive reading after 20 minutes.

## Conclusion

Although rare IgE-mediated reactions to aminoglycosides may occur, skin tests being a useful tool for diagnosis.

## Consent

Written informed consent was obtained from the patient for publication of this abstract and any accompanying images. A copy of the written consent is available for review by the Editor of this journal.

## References

[B1] Schulze S Wollina U Gentamicin-induced anaphylaxis Allergy 2003 58 1 88 9 12580818 10.1034/j.1398-9995.2003.23710_5.x

[B2] Connolly M McAdoo J Bourke JF Gentamicin-induced anaphylaxis Ir J Med Sci 2007 176 317 318 10.1007/s11845-007-0077-z 17724569

